# Automatic Lung Segmentation Based on Texture and Deep Features of HRCT Images with Interstitial Lung Disease

**DOI:** 10.1155/2019/2045432

**Published:** 2019-11-29

**Authors:** Ting Pang, Shaoyong Guo, Xinwang Zhang, Lijie Zhao

**Affiliations:** Center of Network and Information, Xinxiang Medical University, Xinxiang 453000, China

## Abstract

Lung segmentation in high-resolution computed tomography (HRCT) images is necessary before the computer-aided diagnosis (CAD) of interstitial lung disease (ILD). Traditional methods are less intelligent and have lower accuracy of segmentation. This paper develops a novel automatic segmentation model using radiomics with a combination of hand-crafted features and deep features. The study uses ILD Database-MedGIFT from 128 patients with 108 annotated image series and selects 1946 regions of interest (ROI) of lung tissue patterns for training and testing. First, images are denoised by Wiener filter. Then, segmentation is performed by fusion of features that are extracted from the gray-level co-occurrence matrix (GLCM) which is a classic texture analysis method and U-Net which is a standard convolutional neural network (CNN). The final experiment result for segmentation in terms of dice similarity coefficient (DSC) is 89.42%, which is comparable to the state-of-the-art methods. The training performance shows the effectiveness for a combination of texture and deep radiomics features in lung segmentation.

## 1. Introduction

**Figure 1:**
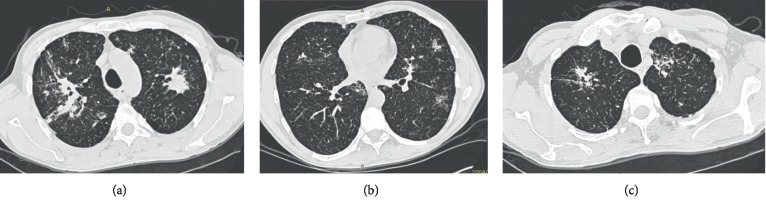
Some examples of lung HRCT with ILD.

Interstitial lung disease (ILD) is a generic term of the clinicopathological entities that are composed by an inhomogeneous group of diseases based on the pathological basic changes of diffuse lung parenchyma, alveolar inflammation, and interstitial fibrosis [1]. It is estimated that the morbidity of ILD is 26–32 cases per 100,000 people per year [2]. Though ILD develops slowly, without early treatment, it may not be eradicated after the breakout, causing great harm to the patients. High-resolution computed tomography (HRCT) can supply such a clear image of the tiny structures of lung tissue so that it is considered as the preferred method to diagnose ILD [3, 4]; the examples are shown in Figure 1. Four main categories of features may be showed at HRCT for ILD: reticular pattern, nodular patterns, increased lung attenuation, and decreased lung attenuation [5, 6].

But due to the capability of radiologists, level of facilities, and nonspecific lung lesion patterns, it also leads to high unpredictability in HRCT interpretations. Computer-aided diagnosis (CAD) system has been widely used to eliminate these defects by quantitative analysis of the characteristics of the pulmonary lesions and by automatic diagnosis. Segmentation of the lung fields in HRCT images into different regions of interest (ROI) is the first step for CAD of lung disease. However, there are challenges now in segmentation of HRCT images for ILD: (1) several noises always occurring in HRCT images resulting in fuzzy edges; (2) depending on low-middle-high level features to distinguish the similar areas; and (3) essential requirements for accuracy of the segmentation algorithm.

Radiomics extracting large amounts of quantitative features from radiographic images plays an important scenario for automatic segmentation [7]. Among the various categories of radiomics features, it can be learned significant information from the ROI through both texture features and deep features for accurate segmentation. Since the texture is formed by the repeated appearance of gray-level distribution in the spatial position, a certain spatial correlation property for the grayscale exists in the image. The gray-level co-occurrence matrix (GLCM) can be used to extract the texture features from abnormal tissues to explain this spatial grayscale relationship [8]. Recently, deep learning as an end-to-end method consisting of multiple neural network layers has been widespread in medical image processing. It can extract deep features using the most popular convolutional neural networks (CNNs) [9].

In this work, we build an automatic segmentation model based on radiomics with deep features and texture features. The contributions of this work are as follows: (1) proposing a new automatic method using the noise preprocessing, deep features, and texture features to make robust lung segmentation and (2) extracting radiomics features to provide support for ILD diagnosis. The rest of this paper is organized as follows: (1) Section 2 reviews some segmentation models used in previous studies. (2) Section 3 describes the proposed method including a detailed process. (3) Section 4 evaluates the feasibility and effectiveness of clinical application for ILD on HRCT images. (4) Section 5 summarizes the research and highlights of the future work.

## 2. Related Works

Lung segmentation methods are mainly divided into four categories [10–12]: threshold methods, edge-based methods, region-based methods, and intelligent methods. The fact that lung looks obviously different from the surrounding regions in CT scans makes the threshold-based methods more easy to understand and operate because of its basic needs that compute a threshold to separate the lung from other tissues [13–16]. However, the main disadvantage of threshold methods is the inaccurate lung segmentation since some of the pulmonary components are similar to the chest structures. The edge-based segmentation functions under edge detector filters at different directions to distinguish the lung boundaries from radiographs [17–19]. Each edge point located by the tracing procedure constitutes a spatially closed outline for the final pulmonary segments. Depending on the fact that adjacent pixels are similar within one region, region-based segmentation is spatially performed by comparing one pixel with the neighbors to ascertain if they belong to the same set. For the region-based methods, the best-known method is the region-growing method [20, 21]. Seed (a small patch) that is first initialized as the most representative voxel continuously grows to extract the target lung region to be segmented [22–24]. Although region-based methods are more efficient than the threshold-based methods, they may need preprocessing and postprocessing when high levels of abnormality are shown in segment regions, for example, noise from CT data. Intelligent methods fuse advanced algorithms in the field of image processing with segmentation, such as pattern recognition [25], fuzzy theory [26], Markov random theory [27], and wavelet analysis [28], which achieves more accurate and realistic results for lung segmentation.

Though these segmentation technologies strive to obtain the final output by defining an initial threshold and combining with other methods to constantly optimize it, no single segmentation method achieves globally optimal performance for all cases.

## 3. The Proposed Automatic Segmentation Method

**Figure 2:**
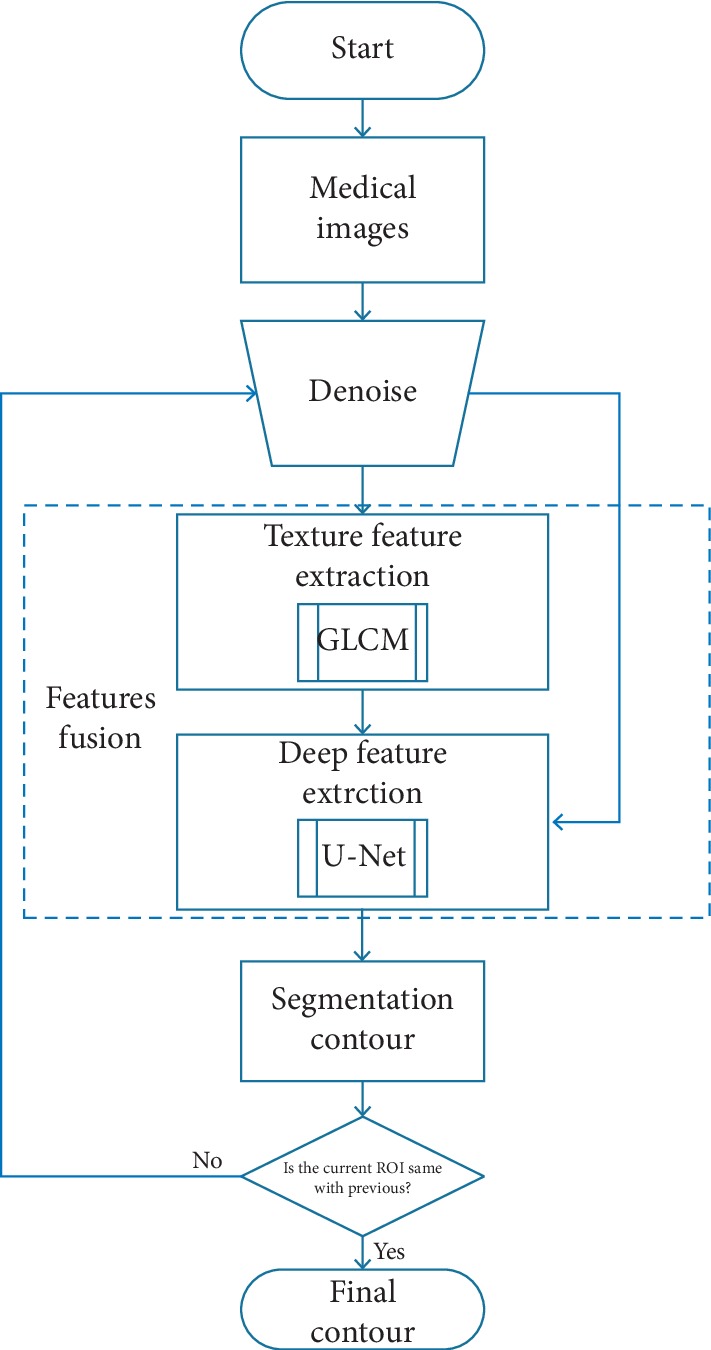
The procedure of the proposed method.

The target of the proposed automatic segmentation model is to accurately segment the lung for ILD. The diagram of the method is shown in Figure 2, and the procedure of the proposed model is preprocessing and segmentation. Preprocessing mainly indicates the denosing, and segmentation focuses on the radiomics features having two stages including texture feature extraction and deep feature extraction. The first stage uses GLCM, of which the input is denosing images and the output is initial segmented images. The second stage uses U-Net [29] (one classic deep learning network), of which the input is denosing images with the output of the first stage and the output is final refined segmented images. The procedure finishes when the segmentation contour is the same with the previous contour.

### 3.1. Preprocessing

The lung graphs for segmentation produced by the machines may add some noise in the process of collection and transmission, leading to the distortion of the HRCT graphs. However, it is very essential to keep the original quality of the radiographs for segmentation to ensure the accuracy of the CAD for ILD. Gaussian noise is the most common noise type caused by the poor light or high temperature in the image. Gaussian noise is a kind of noise whose probability density function obeys Gaussian distribution [30], defined as follows:

(1) $$\begin{array}{c} G\left(x,y\right)=\frac{1}{\sqrt{2\pi \sigma }}e^{\left(-{\left(f\left(x,y\right)-\mu \right)}^{2}\right)/2\sigma^{2}}, \end{array}$$

where *x* and *y* are the position of every pixel on the image, *f*(*x*, *y*) which denotes the original input image is the pixel for every position, *μ* and *σ* , respectively, are the expectation and standard deviation of the noise. After the Gaussian noise is added, the image is defined as follows:

(2) $$\begin{array}{c} S\left(x,y\right)=f\left(x,y\right)+G\left(x,y\right). \end{array}$$

Wiener filter is commonly known as the optimal method for CT image denoise [31]. Meanwhile, Wiener filter is often used to cancel the Gaussian noise and better solve the blurring edge for image segmentation [32–34]. Therefore, in this paper, we employ Wiener filter to reduce the Gaussian noise. Wiener Filter function here is defined by

(3) $$\begin{array}{c} W\left(x,\;y\right)=F\left(S\left(x,y\right)\right)\;H\left(S\left(x,y\right)\right), \end{array}$$

where *F* is the Fourier transform for the input image and *H* is the blurring function. The main principle of Wiener filter is to use the linear estimation to make the mean square error (MSE) between the *W*(*x*, *y*) and *f*(*x*, *y*) minimal, i.e., the Gaussian noise *G*(*x*, *y*) is removed.

### 3.2. Texture Features

The texture features are extracted from the gray-level co-occurrence matrix (GLCM). The GLCM builds the mutual occurrence of different gray levels *i*, *j* between a pair of pixels separated by a certain distance *d* and oriented at a particular direction *θ* in an image space *M* × *N* (ROI) ranging from gray level 0 to *Q* − 1(*Q*=256) [35]. After that, the GLCM element can be defined as follows:

(4) $$\begin{array}{c} q\left(i,j\right)=\left[\left(k,l\right),\left(m,n\right)\right]\left(m\right)-k=x*d,n-l=y*d,I\left(k,l\right)=i,I\left(m,n\right)=j, \end{array}$$

where (*k*, *l*), (*m*, *n*) are the pixels in ROI, *I*(·) is the gray level of the pixel, and 〈·〉 is the number of the pixels which meet the condition. For direction *θ*=0°, 45°, 90°,  and 135°, the values of parameters *x*, *y* at different *θ* are given in Table 1. In this paper, for the texture calculation, the GLCM must be symmetrical, and each entry of the GLCM should be a probability value with a normalization process [36]. The element of the normalized gray-level co-occurrence matrix (NGLCM) is defined as follows:

(5) $$\begin{array}{c} p\left(i,j\right)=\frac{q\left(i,j\right)}{\sum_{i=0}^{Q-1}\sum_{j=0}^{Q-1}q\left(i,j\right)}. \end{array}$$

**Figure 3:**
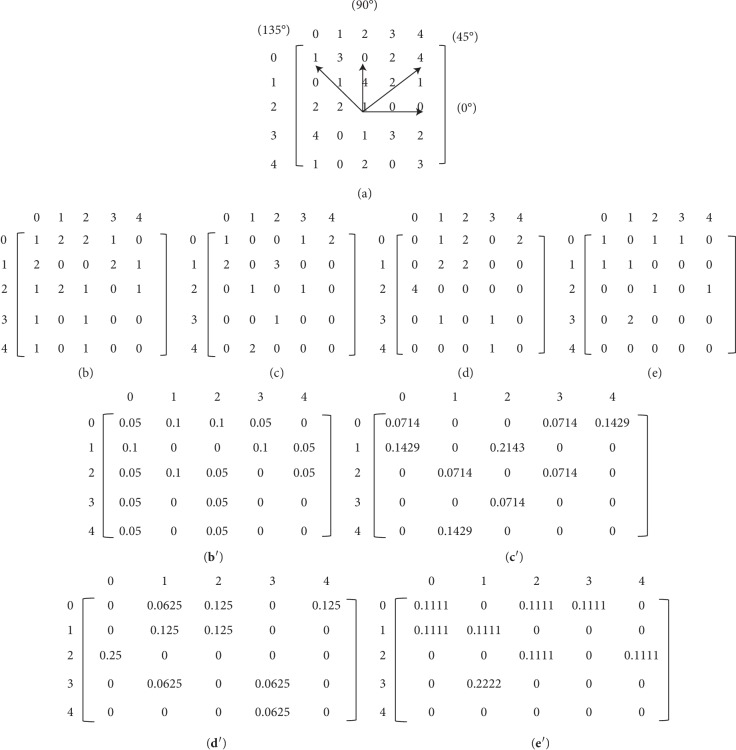
Example of GLCM. (a) The matrix of input image with 5 grayscales. Different distances and orientations such as (b) **d=1**, ***θ=*0**^**°**^, (c) **d=2**, ***θ=*0**^**°**^, (d) **d=1**, ***θ=13*5**^**°**^, and (e) **d=2**, ***θ=13*5**^**°**^. NGLCM calculation: (**b**′)-(b), (**c**′)-(c), (**d**′)-(d), (**e**′)-(e).

Figure 3 shows an example of computation for GLCM and NGLCM where every cell contains the probability value. It can be seen in Figure 3(a) that a 5*∗*5 image including 5 gray levels (from 0 to 4) has a reference pixel (2, 2) with the four directions. For example, the element (0, 2) is 2 in Figure 3(b) as the occurrence of the pair (0, 2) in the input image is 2 at *d*=1 and *θ*=0° according to formula (5). In reference [35], the formulas of 14 features (Angular Second Moment, Contrast, Correlation, Difference Variance, Difference Entropy, etc.) extracted based on GLCM were described in detail. Then, 120 NGLCMs are computed (four directions) and 1680 single values are resulted (14 features).

### 3.3. Deep Features

**Figure 4:**
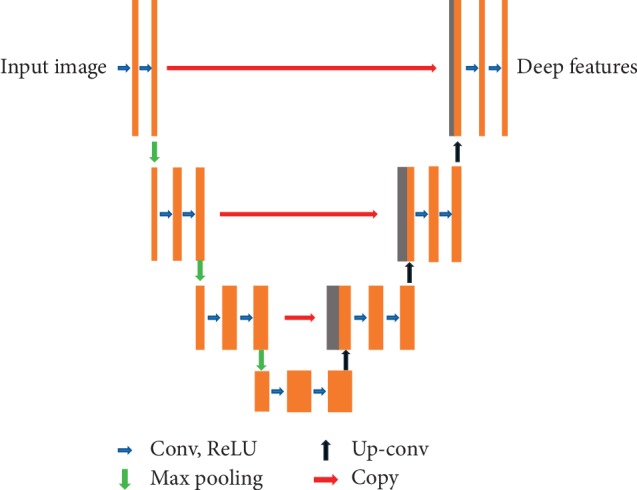
The architecture of U-Net.

The deep CNN features are extracted from the classic U-Net. The U-Net which yields more accurate segmentation is based on the fully convolutional network [37] and suitable for few medical image training.  Figure 4 shows the U-shaped architecture of U-Net. The network consists of two parts, i.e., downsampling and upsampling. In this paper, the downsampling is like an encoder including 3 times of operations with two 3 × 3 convolutional networks followed by a rectified linear unit (ReLU) and a 2 × 2 max pooling layer. Moreover, the upsampling of feature map is a decoder which also consists of 3 times of operations with a 2 × 2 upconvolutional layer followed by a cropping operation from the downsampling, two 3 × 3 convolutional networks, and a ReLU. At every cropping step, one concatenation is added to make up for the loss of border pixels in each convolution. Finally, it obtains a convolutional deep feature map for the segmentation result. The loss function is the combination of softmax and cross-entropy [29]:

(6) $$\begin{array}{c} E=\underset{x}{\sum }\omega \left(x\right)\log\left(\frac{\exp\left(\alpha_{r}\left(x\right)\right)}{\sum_{r\prime =1}^{R}\exp\left(\alpha_{r^{\prime }}\left(x\right)\right)}\right), \end{array}$$

where  *ω*(*x*) is the weight function and *α*_*r*_(*x*) is activation function for the *r* channel.

### 3.4. Algorithm

Based on the overall segmentation architecture with the denoising and feature fusion after training, we can eventually recognize the ROI from the lung area. The detailed steps are illustrated in Algorithm 1.

## 4. Experiments and Discussion

In this section, we validate the method on the medical images for clinical application. First, we introduce the dataset, technical experiment details, and evaluation standard. Then, we, respectively, show and discuss the performance of denoising, segmentation, and training process by comparing with the baseline methods.

### 4.1. Dataset and Technical Details

We experimented on the ILD Database-MedGIFT [38] and selected from 128 patients (47 females and 81males, mean age of 59 years). 108 HRCT image series are stored in DICOM format and reconstructed to 1946 ROIs in PNG format. To have a balance preserving resolution and computational complexity of the models [39], the ROI images here are cropped to pixels 512 × 512, of which 80% (1557) are training data and 20% (389) are testing data. We performed the experiment on the single GPU NVIDIA RTX 2070 using Python language, and CNN was implemented on the framework of TensorFlow, the batch size is 20, the learning rate is 1*e*^−4^, and the epoch is 500. Besides, we see the masks annotated by the database (manual lung segmentation) as ground truth. We adopt dice similarity coefficient (DSC) [40], sensitivity (SEN) [24], and training time (T, one epoch) as evaluation metrics for the proposing method, defined as follows:

(7) $$\begin{array}{c} \text{DSC}=\frac{2*\left|M\cap A\right|\;}{\left|M\right|+\left|A\right|}, \end{array}$$

where *M* is the area of ground truth and *A* is the area of segmentation lung using the proposed method. The value of DSC is between zero and one:

(8) $$\begin{array}{c} \text{SEN}=\frac{\text{TP}}{\text{TP}+\text{FN}}, \end{array}$$

where TP is true positive and FN is false negative.

### 4.2. Segmentation Results

In order to illustrate the effectivity of the proposed method, we compared it with the following methods: (1) GLCM [8], (2) U-Net [29], (3) fully convolutional networks (FCNs) [37] (another commonly used method in segmentation), and (4) GU: GLCM + U-Net (without denoising).

**Figure 5:**
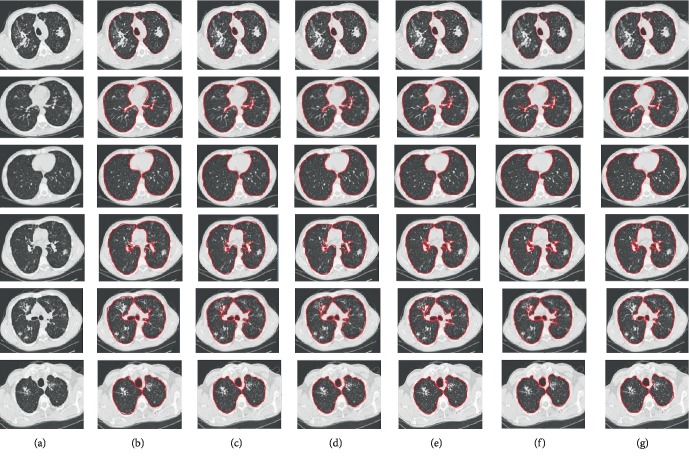
Comparison of segmentation results. (a) Original. (b) GLCM. (c) U-Net. (d) FCN. (e) GU. (f) Ours. (g) Ground truth.

We first show some examples of the segmentation results obtained by the five methods and the ground truth for clarity in Figure 5. We can see from Figure 5 that the achieved segmentation results of our method are the best. Though the results achieved by other methods are similar to ground truth, they often have some false segmented areas. For example, the regions segmented by GU and our method are more accurate, while ours yields slightly better without so much noise. Besides, it can be seen from the second row of Figure 5 that the results received by GLCM, U-Net, and FCN contain some confounding areas.

Then we present the DSC (average value) and SEN of segmentation results on testing dataset with the *T* on training dataset using our method and four compared methods in Table 2. It is significant that our method (in bold) is better than the other four methods all in terms of DSC and SEN. Moreover, the training time of ours is shortest, showing that the complexity is lower and it is easy to perform our method. In particular, the DSC of our method (89.42%) is obviously higher than (80.47%) GLCM which explains that deep features are much more important than texture features for accurate segmentation. On the contrary, the SEN of GLCM is slightly better than U-Net and FCN, which implies that texture features perform better on the problem that much more samples generate accurate segmentation. Hence, the combination of the deep features and texture features is a necessary step in lung segmentation. Besides, U-Net is better than FCN, illustrating that our method can improve the performance by comparing it with the conventional deep learning method.

### 4.3. Influence of Combined Radiomics Strategy

**Figure 6:**
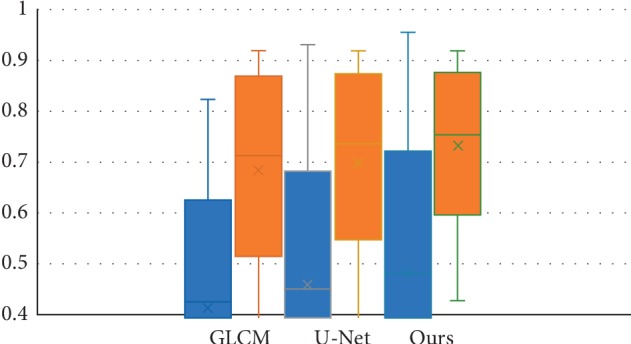
Influence of combined features for segmentation. The blue bar indicates DSC; the orange bar indicates SEN.

In this group of experiment, we illustrate the effectiveness for combination of texture and deep radiomics features in lung segmentation with ILD. We compare the segmentation results of the proposed method in terms of DSC and SEN, respectively, with GLCM, U-Net, and our method, as shown in Figure 6. We can see from Figure 6 that our method is significantly better than using only GLCM or U-Net. U-Net generates much higher DSC value than GLCM, while the SEN value of U-Net is almost the same with GLCM. The combination of the two features promotes better performance.

**Figure 7:**
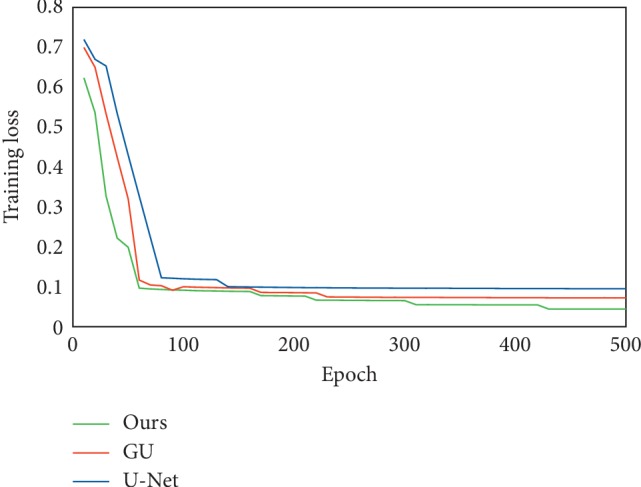
Comparison of the training performance.

We further show about the training performance (according to equation (5)) for U-Net, GU, and ours in Figure 7. The training loss in Figure 7 also shows that our method with lower loss performs better than the other two methods by combining the texture and deep radiomics features.

## 5. Conclusion

We propose a novel automatic segmentation method using radiomics for ILD patterns from HRCT images. After the preprocessing denoising with Wiener filter, we fuse texture features based on GLCM and deep features based on U-Net for the segmentation contour. In the experiments of lung segmentation with ILD, the model reveals higher accuracy and overall performance than the conventional methods. The segmentation results demonstrate both the necessity of denoising and the utility of radiomics features for segmentation. The results of DSC, SEN, and *T* show the usefulness of combination of deep features and texture features. In future, we will try to combine the segmentation model and lung tissue classification for better CAD of ILD.

## Figures and Tables

**Algorithm 1:**
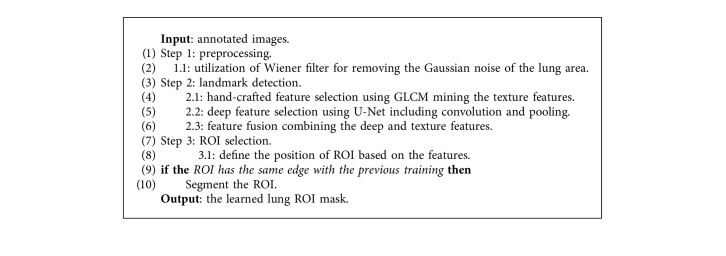
Training for lung ROI selection for HRCT image.

**Table 1:** The values of parameters *x*, *y* at different directions.

*θ*	*x*	*y*
0°	0	1
45°	−1	1
90°	−1	0
135°	−1	−1

**Table 2:** The DSC, SEN, and *T* comparison using different methods.

	GLCM	U-Net	FCN	GU	Ours
DSC (%)	82.47	85.83	83.56	87.16	**89.42**
SEN (%)	93.08	92.94	92.88	94.06	**94.99**
*T* (s)	—	22.3	25.6	21.8	**20.1**

## Data Availability

The HRCT data used to support the findings of this study have been deposited in the ILD Database-MedGIFT repository ([http://medgift.hevs.ch/wordpress/databases/ild-database/]).
